# The effects of increasing body mass index on heartburn severity, frequency and response to treatment with dexlansoprazole or lansoprazole

**DOI:** 10.1111/apt.12270

**Published:** 2013-03-04

**Authors:** D A Peura, B Pilmer, B Hunt, R Mody, M C Perez

**Affiliations:** * University of Virginia Health Sciences CenterCharlottesville, VA, USA; † Takeda Global Research & Development Center, IncDeerfield, IL, USA; ‡ Takeda Pharmaceuticals International, IncDeerfield, IL, USA

## Abstract

**Background:**

Higher body mass index (BMI) is a recognised risk factor for gastro-oesophageal reflux disease (GERD). Data regarding the impact of BMI on proton pump inhibitor (PPI) therapy are conflicting.

**Aim:**

To assess the impact of BMI on baseline heartburn symptom severity and frequency and response to PPI therapy in patients with non-erosive GERD (NERD) or erosive oesophagitis (EO).

**Methods:**

In *post hoc* analyses of phase 3 trial data, 621 NERD and 2692 EO patients were stratified by BMI (<25, 25 to <30 and ≥30 kg/m^2^). NERD patients received either dexlansoprazole MR 30 mg or placebo daily for 4 weeks. EO patients received either dexlansoprazole MR 60 mg or lansoprazole 30 mg for 8 weeks. Symptom frequency and severity were assessed at baseline and subsequently by daily diary.

**Results:**

In both the NERD and EO cohorts, baseline heartburn severity increased with increasing BMI. The impact of PPI therapy on the reduction in heartburn symptom frequency and severity in both NERD and EO patients was similar across BMI categories. EO healing rates in patients treated with dexlansoprazole but not lansoprazole were higher in obese patients compared with those with a BMI <30 kg/m^2^. Differences between the PPIs were small.

**Conclusions:**

The PPIs evaluated in this study reduced the frequency and severity of 24-h heartburn regardless of baseline BMI. In addition, because patients with higher BMI have more severe symptoms at baseline, they may experience greater therapeutic gain with dexlansoprazole (NERD and erosive oesophagitis) and possibly lansoprazole (erosive oesophagitis) treatment.

## Introduction

Gastro-oesophageal reflux disease (GERD) affects up to 20% of Western populations.^1^ Prevalence rates are on the rise,^2^ paralleling increasing rates of overweight [body mass index (BMI) 25 to <30 kg/m^2^] and obese (BMI ≥30 kg/m^2^) adults.^3^ Increasing BMI, both as a categorical and continuous variable, is a strong independent risk factor for more frequent and more severe GERD symptoms and for erosive oesophagitis (EO), and has been linked to complications such as severe EO, Barrett's oesophagus and oesophageal adenocarcinoma.^4^–^7^ Elevated BMI and greater waist circumference are associated with increased intragastric pressure and lower oesophageal pressure; greater likelihood for hiatal hernia; impaired lower oesophageal sphincter function; and more frequent, more prolonged and more proximal episodes of oesophageal acid exposure.^8^–^15^ Among the handful of studies that have assessed the impact of baseline BMI on response to proton pump inhibitor (PPI) therapy in GERD patients, a few have identified higher BMI as a favourable predictor of response, while others have found no effect or a negative effect of BMI on response to PPI therapy.^16^–^23^

The objective of this analysis was to assess differences in baseline heartburn symptom severity and frequency in patients with non-erosive GERD (NERD) or EO when stratified by BMI. In addition, we sought to determine the effect of BMI on heartburn symptom severity and frequency in NERD patients treated with dexlansoprazole MR 30 mg or placebo, and in EO patients treated with dexlansoprazole MR 60 mg or lansoprazole 30 mg.

## Methods

This was a *post hoc* analysis of patients enrolled in phase 3 studies either assessing the efficacy and safety of dexlansoprazole MR 30 mg vs. placebo for 24-h heartburn relief of NERD, or comparing dexlansoprazole MR 60 mg to lansoprazole in EO healing. Table 1 provides the relevant details of each study. Only the approved doses of dexlansoprazole MR for either NERD (30 mg) or EO healing (60 mg) are included in this analysis. Dexlansoprazole MR 30 mg and placebo were administered once daily to 315 and 317 endoscopically confirmed NERD patients, respectively, in a randomised, double-blind, 4-week study.^24^ NERD patients were required to have a ≥6-month history of heartburn, as well as heartburn symptoms ≥4 of 7 days prior to randomisation. In two 8-week, double-blind, randomised healing studies, 2737 endoscopically confirmed EO patients received dexlansoprazole MR 60 mg (*n* = 1374) or lansoprazole 30 mg (*n* = 1363) once daily.^25^ In all studies, the percentage of 24-h heartburn-free days was assessed by twice-daily diary entries and heartburn severity was rated using a 5-point scale (0 = none to 4 = very severe).

**Table 1:** Description of NERD and EO phase 3 studies

	NERD^24^	EO^25^
Inclusion criteria	Men and women ≥18 years of age with a history of heartburn for ≥6 months, experiencing heartburn ≥4 of 7 days preceding randomisation, and normal oesophageal mucosa at screening endoscopy	Men and women ≥18 years of age with endoscopically confirmed EO. Patients with more severe EO (LA grade C and D) were to compose 30% of the study group
Exclusion criteria*	Pregnancy or lactation; active gastric/duodenal ulcers ≤4 weeks of first dose of study drug; coexisting oesophageal disease, including BO; use of PPI, H_2_RA or antacid; known hypersensitivity to PPI; long-term NSAID use	*H. pylori*-positive; pregnancy or lactation; use of any PPI or H_2_RA during screening; chronic NSAID use; coexisting diseases of the oesophagus, including BO; active gastric/duodenal ulcers or acute upper GI haemorrhage ≤4 weeks of first dose of study drug
Study drug	Dexlansoprazole MR 30 mg, *n* = 315	Dexlansoprazole MR 60 mg, *n* = 1374
	Dexlansoprazole MR 60 mg, *n* = 315	Dexlansoprazole MR 90 mg, *n* = 1355
	Placebo, *n* = 317	Lansoprazole 30 mg, *n* = 1363
Study design	Randomised, double-blind, multicentre, placebo-controlled. After 1:1:1 randomisation on Day -1, patients self-administered study drug QD for 4 weeks. Efficacy assessments were conducted at Weeks 2 and 4. Open-label antacid was provided as rescue medication	Randomised, double-blind, multicentre, active-controlled. After 1:1:1 randomisation on Day -1, patients self-administered study drug QD for 8 weeks. Efficacy assessments were conducted at Weeks 4 and 8. Open-label antacid was provided as rescue medication
Primary efficacy endpoint	Percentage of 24-h heartburn-free days during treatment as assessed by daily electronic diary	Percentage of patients who had complete EO healing over 8 weeks as assessed by endoscopy
Secondary and additional efficacy endpoints*	Mean percentage of days without nighttime heartburn over 4 weeks as assessed by daily electronic diary; mean severity of heartburn	Percentage of patients who had complete EO healing over 4 weeks; percentage of subjects with Grade C and E who had complete healing over 4 weeks; percentage of 24-h heartburn-free days; mean severity of heartburn

BO, Barrett's oesophagus; EO, erosive oesophagitis; H_2_RA, histamine-2 receptor antagonist; LA, Los Angeles; MR, modified release; NERD, non-erosive oesophageal reflux disease; NSAID, nonsteroidal anti-inflammatory drugs; PPI, proton pump inhibitor; QD, once daily.

* Not exhaustive; refer to original publications for complete lists.

Patients with heartburn symptom severity data from the diary during baseline (diary entrees within 7 days prior to randomisation) and during treatment were included in this analysis. For each indication (EO and NERD), baseline characteristics and demographics were summarised by baseline BMI group using descriptive statistics. Statistical comparisons among BMI categories (<25, 25 to <30 and ≥30 kg/m^2^) were performed using a Chi-squared test for categorical and anova for continuous data. For each indication (EO and NERD), the mean heartburn symptom severity during baseline and during treatment, the baseline number of days with heartburn symptoms, and the percentage of 24-h heartburn-free days during treatment were summarised by baseline BMI and treatment group using descriptive statistics (median). Within each BMI category, pairwise comparisons between treatment groups for the percentage of 24-h heartburn-free days during treatment were made using a Wilcoxon rank-sum test. Within each treatment group, statistical comparisons were performed among BMI categories for mean heartburn symptom severity during baseline and during treatment, and for the percentage of 24-h heartburn-free days during treatment using a Kruskal–Wallis test. The rate of EO healing at Week 4 and Week 8 was also summarised by baseline BMI and treatment group. For EO healing rates, pairwise comparisons between treatment groups were made using the Cochran–Mantel–Haenszel test, adjusting for Los Angeles (LA) Grade and BMI, while comparisons among BMI categories within a treatment group were made using a Chi-squared test.

## Results

The distribution of patients with baseline BMI of <25, 25 to <30 and ≥30 kg/m^2^ in the NERD study was 267, 296 and 363, respectively, and 821, 1531 and 1669, respectively, in the EO analysis. In the NERD cohort, the majority of patients were women (71.4%), Caucasian (81.5%), consumed alcohol (55.0%) and caffeine (81.0%), and were *Helicobacter pylori* negative (70.1%). The majority of EO patients were men (53.7%), Caucasian (87.0%), consumers of alcohol (56.5%) and caffeine (78.8%) and were *H. pylori* negative (98.1%). Within each cohort (NERD and EO) baseline characteristics and demographics were clinically comparable between treatment groups, as previously reported.^24^, ^25^ Table 2 provides baseline characteristics and demographics listed by BMI category. With increasing baseline BMI, there was a significant trend toward increasing EO severity: LA Grades A/B and C/D were documented for 77.8% and 22.2%, respectively, of EO patients with BMI <25 kg/m^2^; 70.8% and 29.2% of patients with BMI 25 to <30 kg/m^2^; and 67.9% and 32.1% of patients with BMI ≥30 kg/m^2^ (*P* < 0.001).

**Table 2:** Baseline characteristics and demographics by BMI*

	NERD			EO		
		
	BMI, kg/m^2^			BMI, kg/m^2^		
		
	<25	25 to <30	≥30	<25	25 to <30	≥30
Variable	*N* = 267	*N* = 296	*N* = 363	*N* = 821	*N* = 1531	*N* = 1669
Gender, male, *n* (%)	61 (22.8)	115 (38.9)	89 (24.5)	443 (54.0)	941 (61.5)	774 (46.4)
Ethnicity, *n* (%)						
Not Hispanic or Latino	221 (82.8)	226 (76.4)	302 (83.2)	767 (93.4)	1400 (91.4)	1502 (90.0)
Race, *n* (%)						
American Indian/Alaskan Native	0	2 (0.7)	3 (0.8)	6 (0.7)	14 (0.9)	22 (1.3)
Asian	9 (3.4)	6 (2.0)	1 (0.3)	90 (11.0)	66 (4.3)	16 (1.0)
Black/African American	20 (7.5)	42 (14.2)	66 (18.2)	16 (1.9)	55 (3.6)	116 (7.0)
Native Hawaiian/Pacific Islander	0	2 (0.7)	2 (0.6)	1 (0.1)	0	3 (0.2)
Caucasian	234 (87.6)	238 (80.4)	283 (78.0)	692 (84.3)	1355 (88.5)	1451 (86.9)
Multiracial	4 (1.5)	3 (1.0)	6 (1.7)	15 (1.8)	38 (2.5)	56 (3.4)
Unknown	0	3 (1.0)	2 (0.6)	1 (0.1)	3 (0.2)	5 (0.3)
Age, years						
Mean ± s.d.	46.4 ± 15.10	48.9 ± 13.68	47.6 ± 12.92	44.9 ± 16.19	49.5 ± 13.40	47.3 ± 12.39
Range	18–86	18–85	20–76	18–90	18–87	19–84
BMI, kg/m^2^						
Mean ± s.d.	22.3 ± 1.86	27.4 ± 1.39	35.8 ± 5.87	22.6 ± 1.87	27.5 ± 1.41	35.4 ± 5.15
Range	17–25	25–30	30–71	14–25	25–30	30–81
Alcohol drinker, *n* (%)	160 (59.9)	168 (56.8)	181 (49.9)	444 (54.1)	921 (60.2)	907 (54.4)
Smoker, *n* (%)	55 (20.6)	55 (18.6)	58 (16.0)	206 (25.1)	375 (24.5)	386 (23.1)
Caffeine user, *n* (%)	211 (79.0)	230 (77.7)	309 (85.1)	604 (73.6)	1202 (78.6)	1361 (81.6)
*H. pylori* status, *n* (%)						
Positive	68 (25.5)	102 (34.5)	101 (27.8)	14 (1.7)	18 (1.2)	30 (1.8)
Negative	198 (74.2)	191 (64.5)	260 (71.6)	803 (97.8)	1507 (98.4)	1635 (98.0)
Unknown	1 (0.4)	3 (1.0)	2 (0.6)	4 (0.5)	6 (0.4)	4 (0.2)
Baseline Los Angeles Grade of EO, *n* (%)						
A				368 (44.8)	537 (35.1)	511 (30.6)
B	N/A	N/A	N/A	271 (33.0)	547 (35.7)	623 (37.3)
C				152 (18.5)	363 (23.7)	408 (24.4)
D				30 (3.7)	84 (5.5)	127 (7.6)

BMI, body mass index; EO, erosive oesophagitis; N/A, not applicable; NERD, non-erosive gastro-oesophageal reflux disease; s.d., standard deviation.

* All patients with evaluable diary data and heartburn symptom severity data at baseline and during treatment were included in this analysis.

At baseline, symptom severity, as assessed by twice-daily diary entries, increased with increasing BMI in both NERD and EO patients. (Table 3) This trend was statistically significant among EO patients for either treatment group (*P* < 0.001) but not among NERD patients (*P* = 0.483 for dexlansoprazole group; *P* = 0.225 for placebo group). Treatment with dexlansoprazole MR 30 mg in NERD patients and with dexlansoprazole MR 60 mg or lansoprazole 30 mg in EO patients with higher BMI resulted in lower heartburn symptom severity compared with patients with lowest BMI. (Table 3) The trend was statistically significant (*P* = 0.013) only for EO patients treated with dexlansoprazole MR 60 mg, but not for lansoprazole in EO (*P* = 0.074) or dexlansoprazole 30 mg in NERD (*P* = 0.678). Among NERD patients, treatment with dexlansoprazole MR 30 mg led to significantly greater improvements in heartburn symptom severity in every BMI category compared with placebo (*P* ≤ 0.0001). While there were no statistically significant differences between treatment groups in symptom severity for EO patients in any BMI category, the difference between dexlansoprazole MR 60 mg and lansoprazole 30 mg did approach significance (*P* = 0.052) for the highest BMI category (≥30 kg/m^2^).

**Table 3:** Heartburn symptom severity according to twice-daily diary entries at baseline and during treatment

	NERD						EO					
		
	Dexlansoprazole MR 30 mg			Placebo			Dexlansoprazole MR 60 mg			Lansoprazole 30 mg		
				
	BMI, kg/m^2^						BMI, kg/m^2^					
		
Variable (median)	<25	25 to <30	≥30	<25	25 to <30	≥30	<25	25 to <30	≥30	<25	25 to <30	≥30
Baseline 24-h symptom severity	*n* = 89	*n* = 107	*n* = 115	*n* = 100	*n* = 86	*n* = 123	*n* = 273	*n* = 481	*n* = 553*	*n* = 252	*n* = 501	*n* = 549*
	1.14	1.21	1.36	1.32	1.42	1.50	1.07	1.17	1.43	1.00	1.25	1.43
Mean symptom severity during treatment	*n* = 89	*n* = 107	*n* = 115	*n* = 101	*n* = 86	*n* = 123	*n* = 282	*n* = 480	*n* = 556†	*n* = 252	*n* = 517	*n* = 551
	0.50	0.33	0.38	0.80	0.99	0.97	0.19	0.13	0.12	0.20	0.15	0.14

BMI, body mass index; EO, erosive oesophagitis; NERD, non-erosive oesophageal reflux disease.

Severity scale: 0 = none, 1 = mild, 2 = moderate, 3 = severe and 4 = very severe.

Statistically significant differences among BMI categories within treatment group using Kruskal–Wallis test at *P < 0.001; †*P* = 0.013.

**Figure 1:**
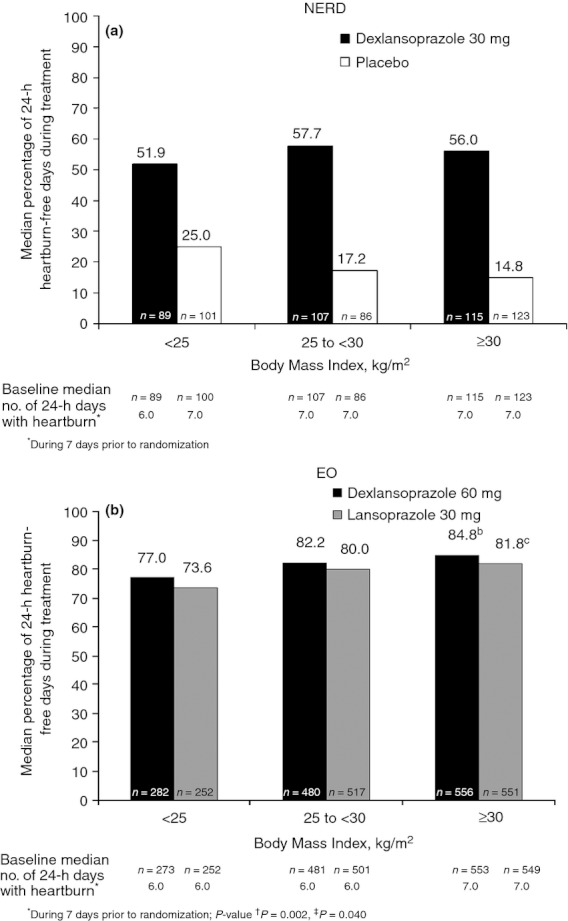
Frequency of 24-h heartburn-free days during treatment in (a) non-erosive oesophageal reflux disease (NERD) and (b) erosive oesophagitis (EO) patients. Statistically significant differences among BMI categories within treatment group were determined using Kruskal–Wallis test.

Median frequency of 24-h days with heartburn at baseline (during the 7 days prior to randomisation) was high for all groups, as shown in Figure 1a and b. NERD or EO patients with higher BMI appeared to have a greater percentage of 24-h heartburn-free days during treatment with either dexlansoprazole MR or lansoprazole compared with NERD and EO patients with lower BMI. (Figure 1a and b) This trend was statistically significant among EO patients receiving either dexlansoprazole MR 60 mg (*P* = 0.002) or lansoprazole 30 mg (*P* = 0.040) but not for NERD patients receiving dexlansoprazole MR 30 mg (*P* = 0.570). When comparing the median frequency of 24-h heartburn-free days in the different BMI categories of EO patients, treatment with dexlansoprazole MR 60 mg led to a significantly higher frequency compared with lansoprazole 30 mg (84.8% vs. 81.8%; *P* = 0.022) at the highest BMI category, but not at lower BMI categories. For NERD patients, treatment with dexlansoprazole MR 30 mg led to a significantly higher frequency of 24-h heartburn-free days compared with placebo in every BMI category (*P* ≤ 0.0001).

At the Week 4 visit, EO healing rates, determined by endoscopy, in patients receiving dexlansoprazole MR 60 mg or lansoprazole 30 mg with BMIs <25, 25 to <30 and ≥30 kg/m^2^ were 66.9%, 64.3% and 71.9% (*P* = 0.030), and 64.0%, 65.3%, and 65.4% (*P* = 0.921) respectively. EO healing rates at Week 8 were 85.9%, 84.7% and 87.7% for patients receiving dexlansoprazole MR 60 mg, and 81.2%, 82.8% and 81.1% for those receiving lansoprazole 30 mg, when stratified by BMI (differences among BMI categories were not statistically significant). Overall, EO healing rates at Week 8 were higher with dexlansoprazole MR 60 mg compared with lansoprazole (*P* = 0.003), but not at Week 4 (*P* = 0.14). Patients receiving dexlansoprazole MR with the highest BMI appear to heal faster than those with the lowest BMI.

## Discussion

This analysis of dexlansoprazole MR treatment in patients with NERD and EO suggests that efficacy for 24-h symptom improvement and EO healing was maintained in patients with higher BMI as compared with those with lower BMI. This is notable because patients with NERD or EO and higher BMI appear to have greater frequency and severity of heartburn symptoms, although this numerical trend was not significant in the NERD cohort. In addition, EO patients had a higher grade of oesophagitis at baseline. Treatment for NERD with dexlansoprazole MR 30 mg or for EO with dexlansoprazole MR 60 mg or lansoprazole 30 mg reduced the frequency and severity of 24-h heartburn regardless of baseline BMI. Because of higher baseline heartburn symptom severity observed in both NERD and EO patients with higher BMI, the therapeutic gain with symptomatic response to PPI therapy may be greater than that observed in patients with the lowest BMI. In addition, based on earlier healing, EO patients with higher BMI may actually experience a greater therapeutic effect with dexlansoprazole MR; although, since most individuals with EO receive 8 weeks of treatment, the clinical importance of this observation is uncertain.

Since higher BMI is known to increase intragastric pressure and compromise lower oesophageal sphincter function,^11^–^14^ which can lead to increased oesophageal acid exposure,^8^, ^11^ higher BMI could lead to more frequent and severe heartburn, which has been observed here in both the NERD and EO cohorts. This is further supported by the results of the longitudinal Progression of Gastro-oesophageal Reflux Disease (ProGERD) study, which followed over 6000 GERD patients.^7^ BMI was significantly associated with more severe and frequent heartburn and regurgitation symptoms, along with the greater likelihood of EO.^7^

Higher response rates to dexlansoprazole MR were observed among those with NERD and higher BMI. 16 also found that NERD patients with higher BMI had better symptom responses after 2 weeks of treatment with lansoprazole 30 mg daily than patients with lower BMI. The higher response rates among those with higher BMI suggest that symptoms in overweight individuals may be indicative of true reflux, rather than functional heartburn, as PPIs are generally not effective against functional heartburn symptoms.^26^ In a prospective study of 100 patients presenting with heartburn and/or regurgitation referred for 24-h pH-impedance monitoring, multivariate analysis demonstrated that failure to resolve symptoms with a PPI was primarily associated with BMI ≤25 kg/m^2^ and the presence of functional dyspepsia, even when acid reflux could be demonstrated.^27^ In a multi-country study designed to mimic the reality of clinical practice, higher BMI was a significant predictor of symptom response in a mixed study population of NERD and EO patients treated with pantoprazole 40 mg daily for 8 weeks.^17^ In contrast, BMI was not found to be a predictor of heartburn resolution among a Swedish cohort of NERD patients who were administered omeprazole 20 mg or esomeprazole 20 mg or 40 mg daily for 4 weeks.^22^

In another study, BMI was found to have no impact on the efficacy of rabeprazole 5 mg or 10 mg daily in a cohort of Japanese NERD patients^28^; however, mean BMI was only ∼23 kg/m^2^ and <25% of subjects had a BMI >25 kg/m^2^. In addition, there was a trend towards better symptom improvement in the higher BMI (>25 kg/m^2^) treatment groups. In comparison, mean BMI in our NERD study population was 29.2 kg/m^2^ and 71.2% had a BMI ≥25 kg/m^2^. In fact, 39.2% had a BMI ≥30 kg/m^2^.

In this study, EO healing rates with lansoprazole 30 mg do not appear to be impacted by BMI, while healing rates with dexlansoprazole MR 60 mg were greater in patients with higher BMI. In addition, a significant trend for improved symptom relief with increasing BMI was observed for those treated with dexlansoprazole MR but not with lansoprazole, although differences were small. Similarly, in a *post hoc* analysis of the clinical efficacy of rabeprazole 20 mg vs. omeprazole 20 mg daily, EO patients with a BMI ≥25 kg/m^2^ seemed to benefit more from rabeprazole as demonstrated by faster onset of symptom relief compared with leaner patients, whereas this was not seen for patients who received omeprazole.^7^ Secondary analyses of data from a randomised, double-blind study comparing the efficacy of esomeprazole 40 mg and pantoprazole 40 mg found that BMI was not a significant predictor of heartburn resolution for either PPI.^21^

While some data, including ours, suggest that PPIs may provide greater therapeutic benefit to patients with higher BMI, other data have shown that higher BMI is a predictor of relapse or therapeutice failure. 29compared clinical characteristics of responders and nonresponders to PPI therapy (daily- and twice-daily dosing) and found a BMI ≥30 kg/m^2^ to be an independent predictor of PPI failure (odds ratio: 3.82; 95% CI: 1.45–10.09; *P* = 0.007). EO patients who had complete resolution of heartburn during full dose PPI and were then stepped-down to maintenance doses of their medications (esomeprazole or pantoprazole) were significantly more likely to remain free of heartburn relapse if their BMI was <30 kg/m^2^ (OR: 1.31; 95% CI: 1.03–1.67; *P* = 0.03).^30^ In a Taiwanese study of LA grade A/B EO patients, higher BMI decreased the rate of sustained symptom response after 8 weeks of therapy with esomeprazole 40 mg daily.^18^ However, in a second study of a Taiwanese cohort of LA grade C/D EO patients treated with esomeprazole 40 mg daily, a BMI of >25 kg/m^2^ decreased the likelihood of endoscopically confirmed healing at 1 month by 2.32-fold.^19^

It is unclear whether our results are specific to dexlansoprazole MR or can be extended to other medications in the class. The conflicting published data regarding the impact of increasing BMI on the efficacy of various PPI therapies may reflect differences in study design, patient populations and outcome measures. In addition, obesity can potentially impact the pharmacokinetics (PK) of a drug, and therefore its efficacy, by modifying its absorption rate, distribution, metabolism and/or excretion, either directly or indirectly via comorbidities.^31^ Therefore, increased adiposity may impact the efficacy of different PPIs in subtly different ways. There are, however, no studies that have explored the impact of various BMIs on any PPI's PK parameters. Conversely, the unique PK characteristics of a PPI may influence its efficacy in patients of varying BMI. Dexlansoprazole MR employs a Dual Delayed Release™ technology, which is designed to provide a dual-peaked PK profile as opposed to the single peak seen with conventional PPIs.^32^ This prolongs the plasma concentration-time profile of dexlansoprazole and provides an extended duration of acid suppression, thereby increasing the mean intragastric pH and the percent of time intragastric pH >4 over 24 h.^32^ Higher BMI is a significant independent predictor of nocturnal heartburn,^33^ and overweight and obese patients can have greater oesophageal acid exposure in a supine position compared with patients with a normal BMI.^15^ The extended duration of acid suppression provided by dexlansoprazole MR may provide benefit for relief of nocturnal heartburn over a single-release PPI when it is taken once daily in the morning.^34^ Additional studies with similar patient populations and similar endpoints are needed to determine if the results reported here are PPI specific.

This study has a number of limitations. First, it is a *post hoc* analysis and the phase 3 trials from which these data were derived were not prospectively designed to examine the impact of BMI on PPI efficacy. Also BMI was not a factor in treatment randomisation. Nonetheless, demographic data (Table 1) show that the distribution of baseline characteristics and demographics were similar across the different BMI categories, suggesting that our results were not influenced by differences in baseline factors. Second, the NERD study was placebo-controlled, so we cannot know how another PPI would have performed in this clinical scenario. The number of NERD patients in each BMI group may also have been too small to show a statistical significance. It may be that, had we observed similar trends in a greater number of NERD patients in each BMI category, statistical significance would have been achieved. Also, NERD patients were not characterised by pH testing or pH-impedance monitoring, so likely the groups included some patients with hypersensitive oesophagus^35^ or functional heartburn.^36^ Finally, heartburn was not an inclusion criteria for the EO studies,^25^ which may have introduced bias, although 85% of subjects did have heartburn symptoms on at least 4 of 7 days during the baseline period.

In conclusion, the data shown here suggest that NERD and EO patients with higher BMI experience more frequent and severe heartburn symptoms. Yet the symptoms can be effectively treated with approved once-daily doses of dexlansoprazole MR. Since patients with higher BMI have greater baseline symptom severity and frequency, and more severe grades of EO, they may actually derive greater therapeutic benefit from dexlansoprazole MR treatment than leaner patients. Additional prospective studies designed to assess the impact of increasing BMI on therapeutic response to PPI therapy in both NERD and EO patients are needed to confirm these results.

## Authorship

*Guarantor of the article*: David A. Peura.

*Author contributions*: David Peura had full access to the data and was involved in the study design and concept, analysis and interpretation of data, drafting of the manuscript, and critical revision of the manuscript for important intellectual content. Barbara Hunt was involved in the study concept and design, generation and statistical analysis of the data, drafting of the manuscript, and critical revision of the manuscript for important intellectual content. Betsy Pilmer, Reema Mody and M. Claudia Perez were involved with study concept and design, analysis and interpretation of data, drafting of the manuscript, critical revision of the manuscript for important intellectual content and they provided final approval to submit the manuscript for publication. All authors approved the final version of the manuscript.
